# Successful treatment of refractory amyopathic dermatomyositis with upadacitinib in prior JAK inhibitor failure

**DOI:** 10.1016/j.jdcr.2024.06.033

**Published:** 2024-07-08

**Authors:** Alice Sohn, Nicole Bouché, George Michael Lewitt, Eingun James Song

**Affiliations:** a Frontier Dermatology, Mill Creek, Washington; b Elson S. Floyd College of Medicine, Spokane, Washington; c Illinois Dermatology Institute, Chicago, Illinois

**Keywords:** biologics, clinical research, dermatomyositis, drug response, general dermatology, immunodermatology, Medical Dermatology, upadacitnib

## Introduction

Dermatomyositis (DM) is a chronic idiopathic inflammatory condition involving the skin and muscles that classically presents with a heliotrope rash and Gottron’s papules.^1^ Intravenous immune globulin is currently the only FDA-approved treatment for DM. Other treatments include photoprotection, topical corticosteroids, antimalarials, and traditional systemic immunosuppressants.^1^ Unfortunately, many patients are treatment refractory, highlighting the need for additional effective and safe therapies.

Recent studies have demonstrated upregulation of the type 1 interferon (IFN) pathway to be associated with DM. Indeed, treatment with IFN beta-1a therapy for multiple sclerosis has been reported to trigger DM,^2^ while inhibiting IFN signaling has been shown to be beneficial in DM.^1^^,^^3^^,^^4^ Because type 1 IFNs signal through the Janus kinase (JAK) pathway, JAK inhibitors have been used off-label to treat DM.^5^ Upadacitinib is an oral JAK-1 (JAK1) inhibitor that has been approved for the treatment of multiple immune-mediated inflammatory diseases including rheumatoid arthritis, psoriatic arthritis, and atopic dermatitis. Herein, we report a case of amyopathic DM (ADM) refractory to baricitinib but experienced significant improvement upon treatment with upadacitinib.

## Case report

**Fig 1:**
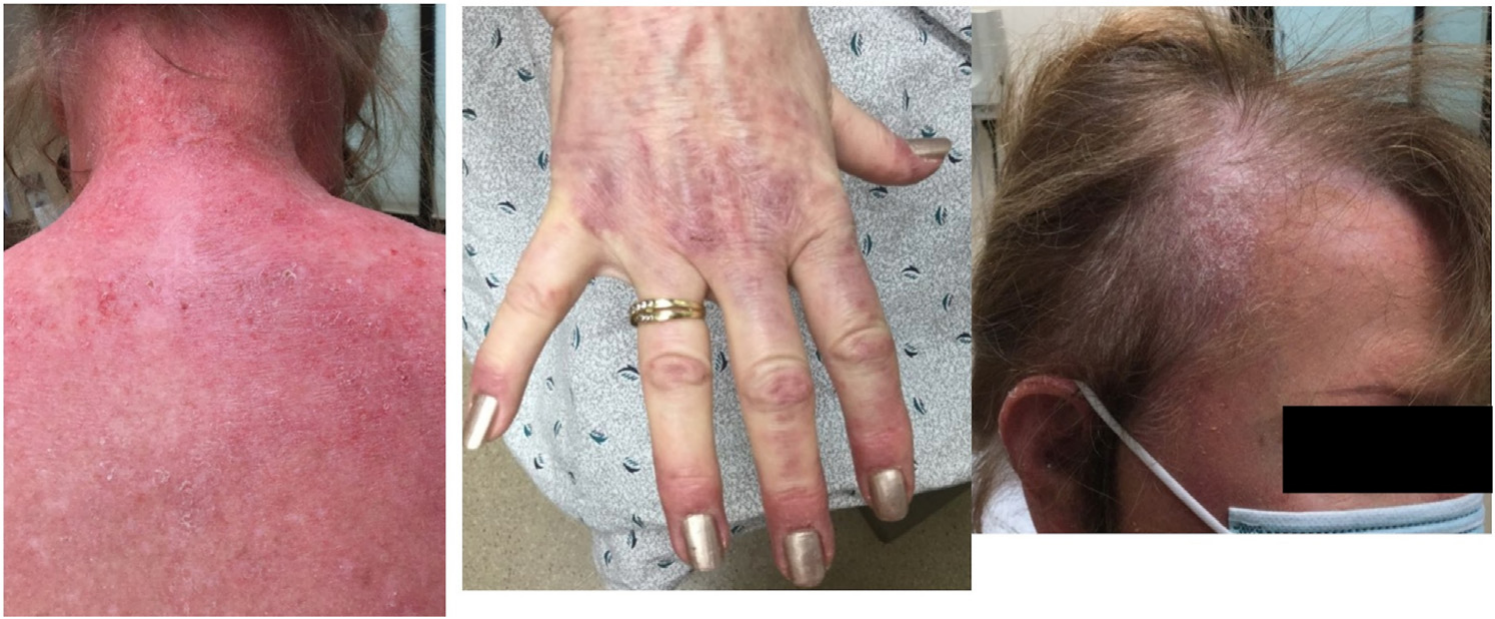
Initial presentation before treatment.

**Fig 2:**
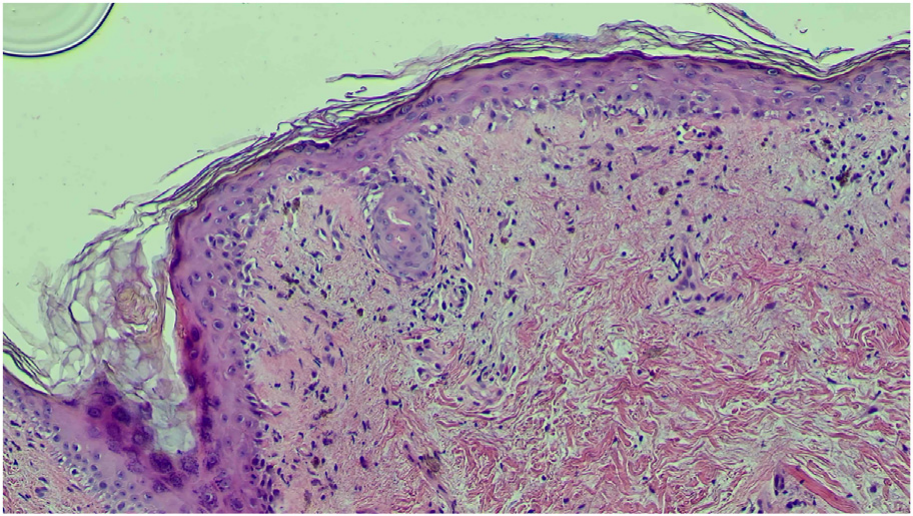
Hematoxylin and eosin (10× magnification) demonstrating a mild lichenoid lymphocytic infiltrate with prominent interface vacuolar change.

A 66-year-old woman presented to our clinic for evaluation of a long-standing, widespread, pruritic eruption involving her scalp, trunk, arms, and legs. On examination, she had erythematous to violaceous psoriasiform patches and plaques involving most of the scalp, neck, chest, back, extensor arms, hands, and thighs (Fig 1). Histopathology showed an interface dermatitis consistent with DM (Fig 2). Her workup was notable for a positive antinuclear antibody 1:160 with a speckled pattern and anti-Jo1 antibody (negative for transcriptional intermediary factor 1-γ, nuclear matrix protein 2, and anti-melanoma differentiation-associated gene 5). Age-appropriate cancer screening was negative and baseline pulmonary function tests were within normal limits. The patient demonstrated 5/5 muscle strength with normal muscle enzymes and was subsequently diagnosed with ADM.

The patient was initially treated with topical corticosteroids, hydroxychloroquine, and methotrexate with minimal improvement. The patient demonstrated the most improvement with baricitinib 4 mg daily, but still had significant itching and skin disease after 3 months of treatment.

**Fig 3:**
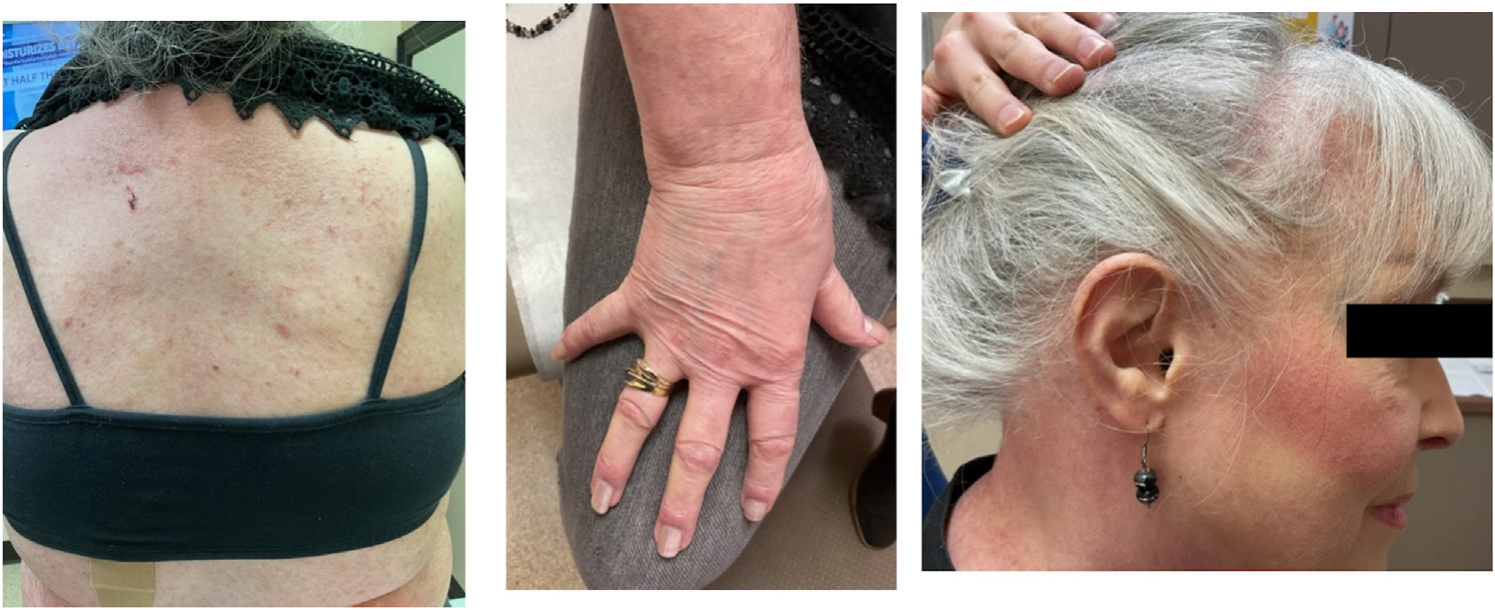
Three months after treatment with upadacitinib 30 mg daily.

Decision was made to try off-label use of upadacitinib 30 mg daily, which resulted in substantial improvement in skin clearance and itch reduction following 3 months of treatment (Fig 3). Patient tolerated the medication well without any notable laboratory changes. As of today’s writing, the patient has been on upadacitinib for 6 months and continues to be almost clear.

## Discussion

Enhanced type 1 IFN signaling has been shown to be involved in a number of autoimmune diseases, including DM.^4^^,^^6^ Given the obligate role of the JAK pathway in IFN signaling, JAK inhibitors have been used off-label to treat DM, with tofactinib and baricitinb being the most frequently reported.^1^ Brepocitinib, a dual tyrosine kinase 2 and JAK1 inhibitor, is currently being evaluated in phase III clinical trials for adults with DM and is poised to be the first JAK inhibitor approved for DM.^7^

While there is less published data with upadacitinib, Beckett *et al* recently reported a case series of 10 patients with myositis who were successfully treated with upadacitinib (5 patients with classic DM, 3 with ADM, and 2 with antisynthetase syndrome). Those diagnosed with classic DM and ADM experienced significant improvement of their cutaneous symptoms upon treatment with upadacitinib.^8^

Our patient’s prior failure to baricitinib highlights the inherent differences among therapies even within the same class. Differences in JAK selectivity, the mode of target binding, drug metabolism, and tissue penetration may lead to differences in the clinical profile of a particular JAK inhibitor.^9^ Because type 1 IFN signaling is primarily mediated by JAK1/tyrosine kinase 2, selective JAK1 inhibitors such as upadacitinib may be more effective in DM.^10^ Furthermore, individual variations such as single-nucleotide polymorphisms that can affect signal transducer and activation of transcription isoforms and differential JAK expression at sites of inflammation may also account for treatment response differences.^11^ Indeed, studies have shown in rheumatoid arthritis that prior JAK inhibitor failure should not preclude switching to another JAK inhibitor.^12^

Being a single case report, there are limitations including sample size and short duration of follow-up. As with most cases of off-label usage, our patient’s insurance would not approve upadacitinib for her indication and therefore has been managed with samples. Longer-term data will be of utmost importance given the paraneoplastic association with DM and the increased risk of malignancy seen in high-risk rheumatoid arthritis patients treated with oral tofacitinib.^13^

## Conflicts of interest

Dr Lewitt reports relationship with AbbVie, Amgen, Lilly, Janssen, Orthodermatologics, UCB, Novartis, Pfizer, Dermavant, Bristol Myers Squibb, Incyte, Arcutis, Sol-Gel, AoBiome, Galderma Laboratories, Leo, Novan, Derm-Tech, and Dermata-Therapeutics; Dr Song reports relationship with BMS, AbbVie, Eli Lilly, Janssen, Novartis, UCB, Pfizer, Amgen, Dermavant, Arcutis, Incyte, SUN, Boehringer Ingelheim, Sanofi & Regeneron, and Ortho-dermatologics. Drs Sohn and Bouché have no conflicts of interest to declare.
